# Supplementary material for: Antigenic assessment of the H3N2 component of the 2019-2020 Northern Hemisphere influenza vaccine
Source: Nat Commun. 2020 May 15;11:2445. doi: 10.1038/s41467-020-16183-y (PMC7229105; doi:10.1038/s41467-020-16183-y)
Supplement: Supplementary file 2 — Reporting Summary [file 41467_2020_16183_MOESM2_ESM.pdf]

nature research

Corresponding author(s): Scott E. Hensley

Last updated by author(s): Feb 3, 2020

Reporting Summary

Nature Research wishes to improve the reproducibility of the work that we publish. This form provides structure for consistency and transparency in reporting. For further information on Nature Research policies, see Authors & Referees and the Editorial Policy Checklist .

Statistics

For all statistical analyses, confirm that the following items are present in the figure legend, table legend, main text, or Methods section.

n/a	Confirmed	
☐	☒	The exact sample size (n) for each experimental group/condition, given as a discrete number and unit of measurement
☐	☒	A statement on whether measurements were taken from distinct samples or whether the same sample was measured repeatedly
☐	☒	The statistical test(s) used AND whether they are one- or two-sided<br>*Only common tests should be described solely by name; describe more complex techniques in the Methods section.*
☒	☐	A description of all covariates tested
☐	☒	A description of any assumptions or corrections, such as tests of normality and adjustment for multiple comparisons
☐	☒	A full description of the statistical parameters including central tendency (e.g. means) or other basic estimates (e.g. regression coefficient) AND variation (e.g. standard deviation) or associated estimates of uncertainty (e.g. confidence intervals)
☐	☒	For null hypothesis testing, the test statistic (e.g. F, t, r) with confidence intervals, effect sizes, degrees of freedom and P value noted<br>*Give P values as exact values whenever suitable.*
☒	☐	For Bayesian analysis, information on the choice of priors and Markov chain Monte Carlo settings
☒	☐	For hierarchical and complex designs, identification of the appropriate level for tests and full reporting of outcomes
☒	☐	Estimates of effect sizes (e.g. Cohen's d, Pearson's r), indicating how they were calculated

Our web collection on statistics for biologists contains articles on many of the points above.

Software and code

Policy information about availability of computer code

Data collection	No software was used for data collection.
Data analysis	GraphPad Prism version 8, R version 3.5.3

For manuscripts utilizing custom algorithms or software that are central to the research but not yet described in published literature, software must be made available to editors/reviewers. We strongly encourage code deposition in a community repository (e.g. GitHub). See the Nature Research guidelines for submitting code & software for further information.

Data

Policy information about availability of data

All manuscripts must include a data availability statement. This statement should provide the following information, where applicable:

- Accession codes, unique identifiers, or web links for publicly available datasets
- A list of figures that have associated raw data
- A description of any restrictions on data availability

Source data for figure 1B-E and figure 2 are provided in the Source Data file. The crystal structure of the HA trimer of A/Victoria/361/2011 is available via the Protein Data Bank (PDB accession code 4O5I).

Field-specific reporting

Please select the one below that is the best fit for your research. If you are not sure, read the appropriate sections before making your selection.

☒ Life sciences ☐ Behavioural & social sciences ☐ Ecological, evolutionary & environmental sciences

Life sciences study design

All studies must disclose on these points even when the disclosure is negative.

Sample size	Based on a previous study (Gouma et al., 2019), we estimated that the coefficient of variation (CV) of log2-transformed antibody titers in ferrets should be less than 20%. With 3 ferrets per group, a twofold difference in antibody titers with an alpha of 0.05 gives >95% power.<br>In a large serosurvey that we conducted with human serum samples, CV of log2-transformed antibody titers in subjects 18-66 years of age was 30%. With 62 subjects, a twofold difference in antibody titers with an alpha of 0.05 gives >95% power.
Data exclusions	No data were excluded.
Replication	Each ferret sample was tested in 3 independent FRNTs and each human sera was tested in 2 independent FRNTs. All attempts at replication were successful. Geometric mean titers of the replicates were used for analysis.
Randomization	Randomization was not applicable for the human study. All participants received a seasonal influenza vaccine, and there was not an unvaccinated group.
Blinding	Blinding was not applicable during sample collection, since sample collection only involved blood draws from participants before and after seasonal influenza vaccination. Samples and additional participant information were deidentified prior to analysis.

Reporting for specific materials, systems and methods

We require information from authors about some types of materials, experimental systems and methods used in many studies. Here, indicate whether each material, system or method listed is relevant to your study. If you are not sure if a list item applies to your research, read the appropriate section before selecting a response.

Materials & experimental systems

n/a	Involved in the study	
☐	☒	Antibodies
☐	☒	Eukaryotic cell lines
☒	☐	Palaeontology
☐	☒	Animals and other organisms
☐	☒	Human research participants
☒	☐	Clinical data

Methods

n/a	Involved in the study	
☒	☐	ChIP-seq
☒	☐	Flow cytometry
☒	☐	MRI-based neuroimaging

Antibodies

Antibodies used	anti-NP monoclonal antibody IC5-1B7 (product number NR-43899; BEI Reagent Resources), peroxidase-conjugated goat affinity purified antibody to mouse IgG (product number 855563; MP Biomedicals)
Validation	IC5-1B7 was verified to be NP-specific via western blot (BEI Reagent Resources web page).<br>Specificity: NP (folded and misfolded) from human influenza A virus<br>Immunizing antigen: cells infected with human influenza A virus<br>Publications: PMID 24971535 , PMID 29109276 , PMID 31400756, PMID 31598646<br><br>According to the manufacturer's datasheet, the anti-mouse antibody is suitable for use as a reagent in enzyme immunoassays, cell and tissue staining, cell and tissue labeling, and blot immunostaining. The antibody titer is standardized by microtiter plate ELISA with mouse IgG. The product is tested for purity and specificity at final concentration by immunoelectrophoresis. The antibody is goat IgG; no trace of albumin is detected. It shows reactivity to mouse IgG; cross-reactivity to other species may exist. Because the product is directed against whole IgG, some antibodies are expected to bind light chain sites common to all immunoglobulins. Antibody activity to non-immunoglobulin serum proteins is not present.

Eukaryotic cell lines

Policy information about cell lines

Cell line source(s)	Madin-Darby Canine Kidney-Siat 1 cells were used for FRNTs. These cells were obtained from Fred Hutchinson Cancer Center.
Authentication	The MDCK-SIAT1 cell line was not authenticated but these cells are routinely used in our laboratory for influenza virus assays.
Mycoplasma contamination	Cell lines tested negative for mycoplasma contamination.

Commonly misidentified lines (See ICLAC register)	No commonly misidentified cell lines were used in the study.

Animals and other organisms

Policy information about studies involving animals; ARRIVE guidelines recommended for reporting animal research

Laboratory animals	Post-infection sera from 6 male ferrets was used in this study.
Wild animals	The study did not involve wild animals.
Field-collected samples	The study did not involve samples collected from the field.
Ethics oversight	Ferret experiments were completed under an Institutional Animal Care and Use Committee-approved protocol at Noble Life Sciences (Gaithersburg, MD).

Note that full information on the approval of the study protocol must also be provided in the manuscript.

Human research participants

Policy information about studies involving human research participants

Population characteristics	We collected serum samples from 62 humans, including 44 females and 18 males. The median age of the participants in our study was 34 years (range 18-66 years).
Recruitment	Subjects were recruited via placement of posters and flyers in key locations on the campus of the University of Pennsylvania, via Penn Medicine Intranet, and via emails that were sent to students, faculty and staff. Subjects were enrolled based on year of birth, resulting in a wide age range (18-66 years). Since all participants were affiliated with the University of Pennsylvania, it is unclear if our study participants are fully representative of the entire population within Philadelphia.
Ethics oversight	The study was approved by the institutional review board of the University of Pennsylvania.

Note that full information on the approval of the study protocol must also be provided in the manuscript.
