# Supplementary material for: Antigenic assessment of the H3N2 component of the 2019-2020 Northern Hemisphere influenza vaccine
Source: Nat Commun. 2020 May 15;11:2445. doi: 10.1038/s41467-020-16183-y (PMC7229105; doi:10.1038/s41467-020-16183-y)
Supplement: Supplementary file 1 — Peer Review File [file 41467_2020_16183_MOESM1_ESM.pdf]

Editorial Note: This manuscript has been previously reviewed at another journal that is not operating a transparent peer review scheme. The manuscript was considered suitable for publication without further review at Nature Communications.
